# Supplementary material for: COVID-19 Vaccine Perceptions among Ebola-Affected Communities in North Kivu, Democratic Republic of the Congo, 2021
Source: Vaccines (Basel). 2023 May 11;11(5):973. doi: 10.3390/vaccines11050973 (PMC10223943; doi:10.3390/vaccines11050973)
Supplement: Supplementary file 1 [file vaccines-11-00973-s001.zip › vaccines-2358584-supplementary.docx]

**Supplemental Figure S1.** Map of health zones (Beni, Butembo, Mabalako) surveyed in North Kivu, The Democratic Republic of the Congo, 2021.


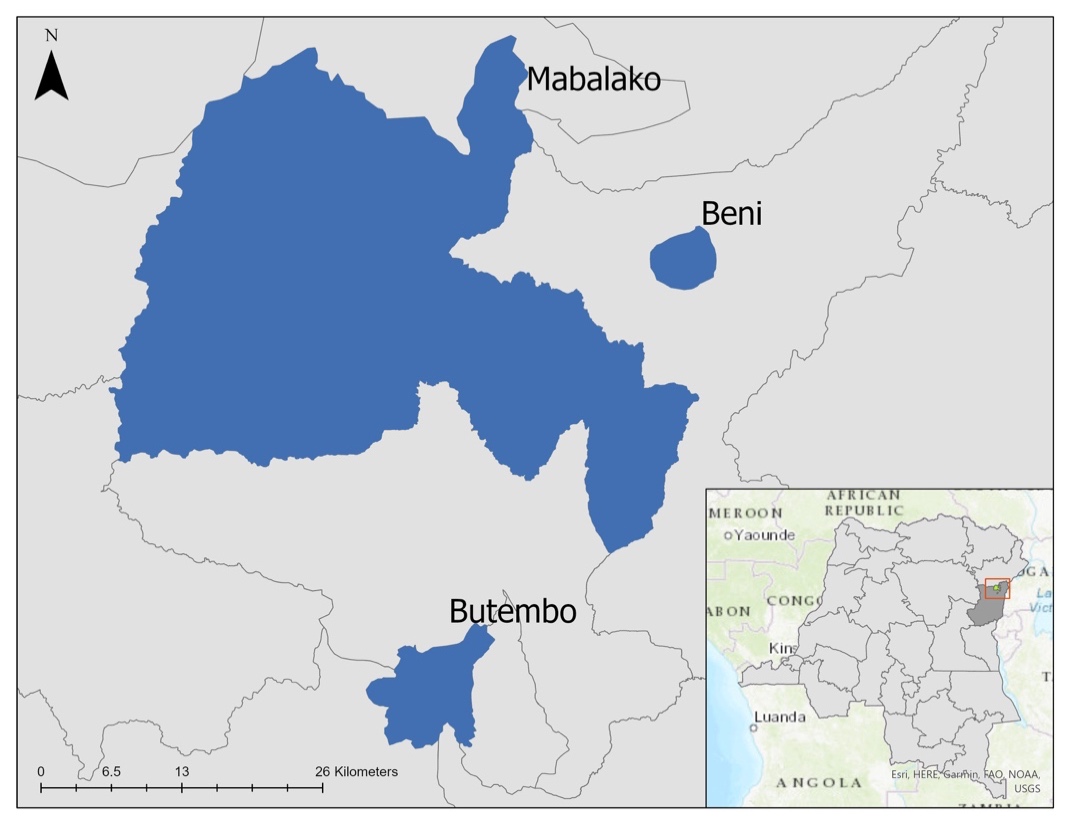


**Supplemental Figure S2.** Survey flow diagram.

**
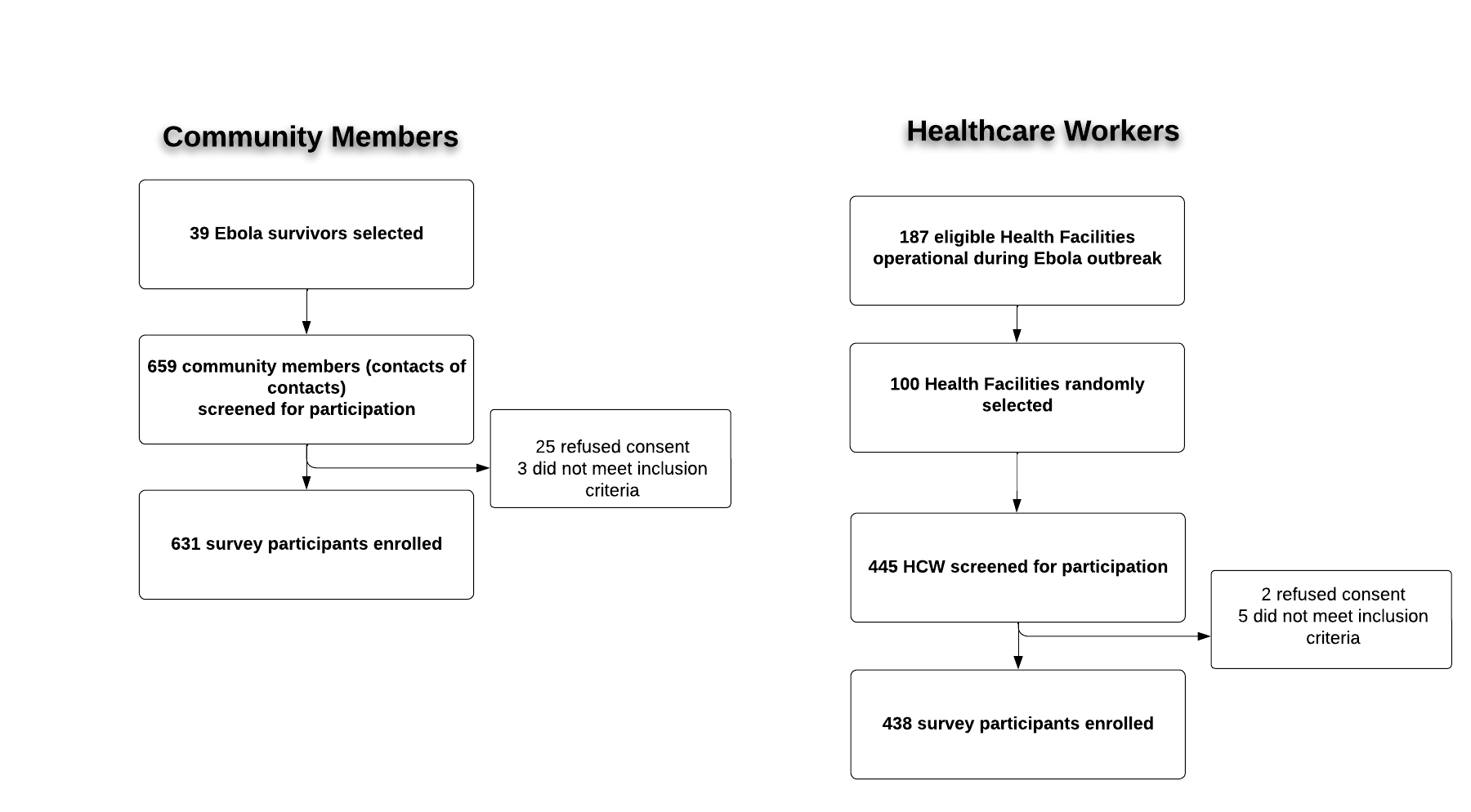
**

**Supplemental Table S1.** Perceptions regarding COVID-19 vaccines among community members (CMs; N=631) and healthcare workers (HCWs; N=438), North Kivu, The Democratic Republic of the Congo, 2021.

| **Questionnaire Item** | **Group** | **Strongly**  **Agree** | **Agree** | **Neutral** | **Disagree** | **Strongly**  **Disagree** | **Unsure/**  **Decline** |
| --- | --- | --- | --- | --- | --- | --- | --- |
| n (%)  [95% CI]^1^ | | | | | | | |
| A COVID-19 vaccine is needed in addition to barrier measures to stop the spread of COVID-19 | CMs | 136 (21.6) | 103 (16.3) | 118 (18.7) | 92 (14.6) | 75 (11.9) | 107 (17) |
|  | HCWs | 101 (23.1)  [20.72, 5.6] | 110 (25.1)  [23.1, 27.3] | 45 (10.3)  [8.9, 11.8] | 89 (20.3)  [18.2, 22.6] | 45 (10.3)  [8.9, 11.8] | 48 (11.0)  [9.4, 12.7] |
| Once vaccinated we do not need barrier measures | CMs | 19 (3.0) | 25 (4.0) | 71 (11.3) | 206 (32.7) | 214 (33.9) | 96 (15.2) |
|  | HCWs | 6 (1.4)  [0.9, 2.1] | 8 (1.8)  [1.3, 2.6] | 19 (4.3)  [3.5, 5.4] | 179 (40.9)  [38.0, 43.8] | 190 (43.4)  [40.5, 46.3] | 36 (8.2)  [6.8, 9.9] |
| Barrier measures are sufficient. A vaccine is not needed | CMs | 69 (10.9) | 70 (11.1) | 93 (14.7) | 167 (26.5) | 139 (22.0) | 93 (14.7) |
|  | HCWs | 43 (9.8)  [8.5, 11.3] | 55 (12.6)  [10.9, 14.4] | 45 (10.3)  [8.9, 11.9] | 141 (32.2)  [29.8, 34.7] | 123 (28.1)  [25.4, 30.9] | 31 (7.1)  [5.9, 8.5] |
| A COVID-19 vaccine should be given to people who are more at risk for severe illness, only^2^ | CMs | 27 (4.3) | 48 (7.6) | 108 (17.1) | 177 (28.1) | 153 (24.3) | 118 (18.7) |
|  | HCWs | 13 (3.0)  [2.2, 4.0] | 33 (7.5)  [6.2, 9.1] | 65 (14.8)  [12.8, 17.2] | 135 (30.8)  [28.4, 33.4] | 136 (31.1)  [28.1, 34.1] | 56 (12.8)  [11.0, 14.8] |
| A COVID-19 vaccine should be given to everyone, not just those who are more at risk for severe illness | CMs | 174 (27.6) | 114 (18.1) | 108 (17.1) | 74 (11.7) | 61 (9.7) | 100 (15.6) |
|  | HCWs | 152 (34.7)  [31.6, 38.0] | 111 (25.3)  [23.1, 27.7] | 48 (11.0)  [9.1, 13.1] | 46 (10.5)  [9.1, 12.0] | 27 (6.2)  [5.1, 7.4] | 54 (12.3)  [10.8, 14.1] |

^1^ 95% CI presented for HCWs as survey methods were used for HCW data analysis based on health facility clustering.

^2^ For example: elderly, health and other essential workers, people with chronic health conditions etc.

**Supplemental Table S2.** General vaccine confidence among community members (N=631) and healthcare workers (N=438), North Kivu, The Democratic Republic of the Congo, 2021.

|  | **Respondent Group** | **Very much** | **Somewhat** | **Very Little** | **Not At All** | **Unsure /**  **Declined** |
| --- | --- | --- | --- | --- | --- | --- |
| Item |  | n(%)  [95% CI]* | | | | |
| How much do you think that vaccines are good? | CM | 240 (38.0) | 220 (34.9) | 72 (11.4) | 40 (6.3) | 59 (9.4) |
|  | HCW | 258 (58.9)  [55.7, 62.1] | 130 (29.7)  [27.1, 32.4] | 27 (6.2)  [5.1, 7.4] | 7 (1.6)  [1.1, 2.4] | 16 (3.7)  [2.8, 4.8] |
| How much do you think that vaccines are safe? | CM | 170 (26.9) | 209 (33.1) | 33 (21.1) | 54 (8.6) | 65 (10.3) |
|  | HCW | 190 (43.4)  [40.5, 46.3] | 168 (38.4)  [35.8, 41.0) | 36 (8.2)  [6.9, 9.7] | 17 (3.9)  [3.0, 5.0) | 27 (6.2)  [5.0, 7.5] |
| How much do you think that vaccines protect against diseases? | CM | 255 (40.4) | 233 (36.9) | 77 (12.2) | 25 (4.0) | 41 (6.5) |
|  | HCW | 256 (58.4)  [55.5, 61.3] | 129 (29.5)  [27.0, 32.0] | 27 (6.2)  [5.1, 7.5] | 14 (3.2)  [2.4, 4.2] | 12 (2.7)  [2.1, 3.6] |
| To which extent do religious leaders in your community approve of vaccination | CM | 314 (49.8) | 154 (24.4) | 50 (7.9) | 14 (2.2) | 99 (15.7) |
|  | HCW | 236 (53.9)  [50.8, 56.9] | 106 (24.2)  [21.9, 26.7] | 25 (5.7)  [4.3, 7.5] | 14 (3.2)  [2.4, 4.2] | 57 (13.0)  [10.9, 15.5] |
| How much do other leaders in this community approve of vaccination? | CM | 290 (46.0) | 186 (29.5) | 42 (6.7) | 17 (2.7) | 96 (15.2) |
|  | HCW | 228 (52.1)  [49.1, 55.0] | 118 (26.9)  [24.6, 29.3] | 21 (4.8)  [3.8, 6.0] | 11 (2.5)  [1.9, 3.4] | 60 (13.7)  [11.4, 16.4] |
|  |  | **Positively** | **Mixed** | **Negatively** | **-** | **Don’t Know/**  **Declined** |
| How do people in this community usually speak about vaccination? | CM | 84 (13.3) | 354 (56.1) | 136 (21.6) | - | 57 (9.0) |
|  | HCW | 45 (10.3)  [8.5, 12.3] | 267 (61.0)  [58.2, 63.7] | 109 (24.9)  [22.5, 27.5] | - | 17 (3.9)  [3.0, 4.9] |
|  | Median [IQR] | | | | | |
| Total Vaccine Confidence Composite Score | CMs | 12 [9–15] | | | | |
|  | HCWs | 14 [11–16] | | | | |

**Supplemental Table S3.** Information important for vaccine-related decisions among community members (CMs) and healthcare workers (HCWs), North Kivu, The Democratic Republic of the Congo, 2021.

|  | **CMs** |  | **HCWs** | |
| --- | --- | --- | --- | --- |
|  | **n**  **(N=631)** | **%** | **n**  **(N=438)** | **% (95% CI)^1^** |
| **What information is important to help you decide to get vaccinated?^2^** |  |  |  |  |
| Side Effects | 285 | 45.2 | 209 | 47.7 (44.4, 51.0) |
| Cost | 25 | 4.0 | 22 | 94.9 (93.7, 96.0) |
| Location where vaccine is offered | 117 | 18.5 | 96 | 21.9 (19.6, 24.4) |
| Vaccine efficacy | 404 | 64.0 | 318 | 72.6 (69.8, 75.2) |
| Eligibility information | 201 | 31.9 | 209 | 47.7 (44.4, 51.0) |
|  | | | | |

^1^ 95% CI presented for HCWs as survey methods used for HCW data analysis

^2^ Multiple selections allowed therefore proportions do not sum to 100%

Abbreviations: CI, confidence interval
