# Supplementary material for: COVID-19 Vaccine Perceptions among Ebola-Affected Communities in North Kivu, Democratic Republic of the Congo, 2021
Source: Vaccines (Basel). 2023 May 11;11(5):973. doi: 10.3390/vaccines11050973 (PMC10223943; doi:10.3390/vaccines11050973)
Supplement: Supplementary file 1 [file vaccines-11-00973-s001.zip › vaccines-2358584-supplementary.pdf]

Supplemental Figure S1. Map of health zones (Beni, Butembo, Mabalako) surveyed in North Kivu, The Democratic Republic of the Congo, 2021.

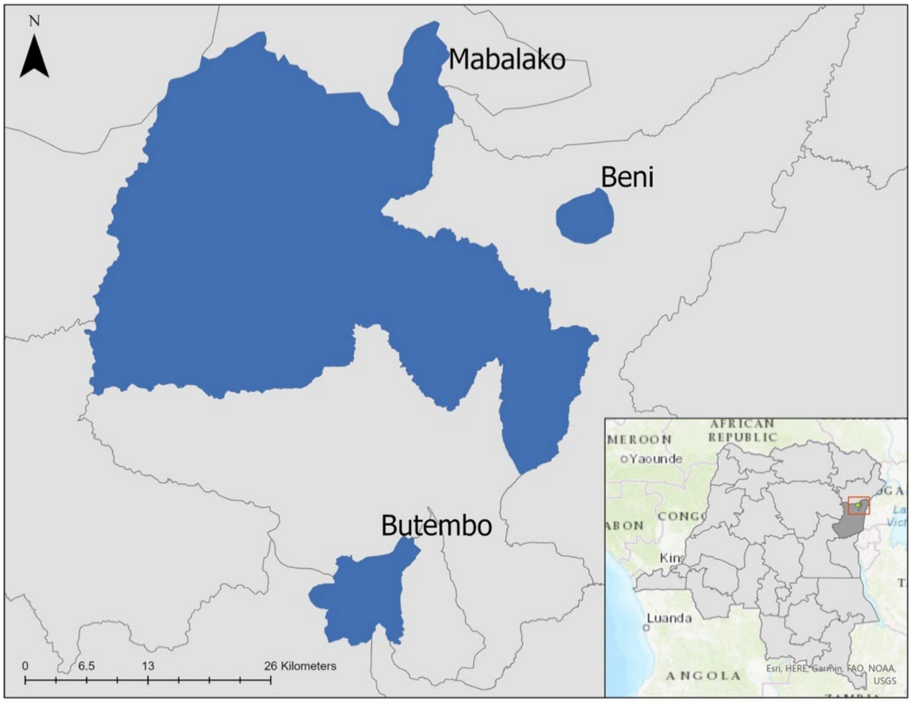

Supplemental Figure S2. Survey flow diagram.

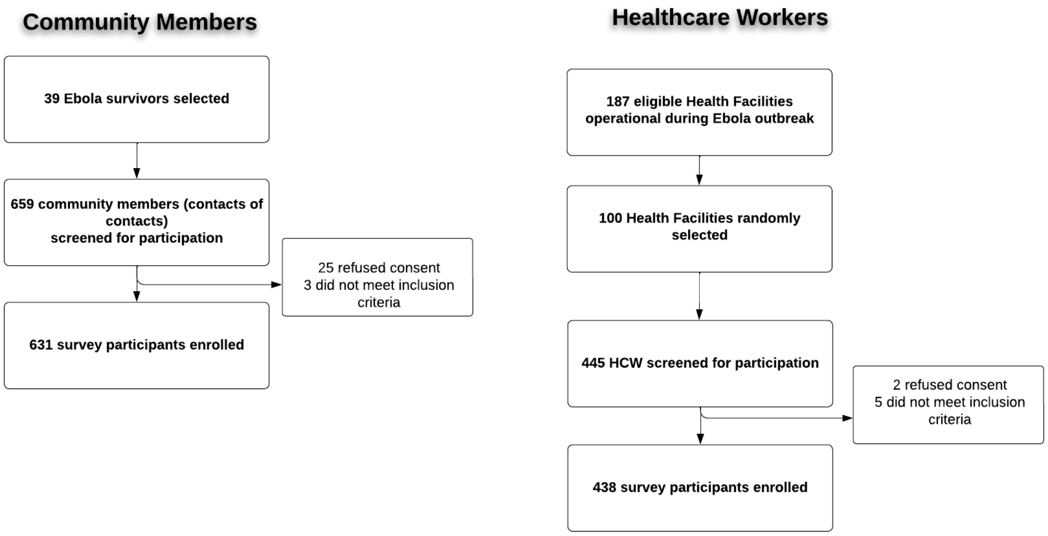

Supplemental Table S1. Perceptions regarding COVID-19 vaccines among community members (CMs; N=631) and healthcare workers (HCWs; N=438), North Kivu, The Democratic Republic of the Congo, 2021.

Questionnaire Item	Group	Strongly Agree	Agree	Neutral	Disagree	Strongly Disagree	Unsure/ Decline
		n (%) [95% CI][1]					
A COVID-19 vaccine is needed in addition to barrier measures to stop the spread of COVID-19	CMs	136 (21.6)	103 (16.3)	118 (18.7)	92 (14.6)	75 (11.9)	107 (17)
	HCWs	101 (23.1) [20.72, 5.6]	110 (25.1) [23.1, 27.3]	45 (10.3) [8.9, 11.8]	89 (20.3) [18.2, 22.6]	45 (10.3) [8.9, 11.8]	48 (11.0) [9.4, 12.7]
Once vaccinated we do not need barrier measures	CMs	19 (3.0)	25 (4.0)	71 (11.3)	206 (32.7)	214 (33.9)	96 (15.2)
	HCWs	6 (1.4) [0.9, 2.1]	8 (1.8) [1.3, 2.6]	19 (4.3) [3.5, 5.4]	179 (40.9) [38.0, 43.8]	190 (43.4) [40.5, 46.3]	36 (8.2) [6.8, 9.9]
Barrier measures are sufficient. A vaccine is not needed	CMs	69 (10.9)	70 (11.1)	93 (14.7)	167 (26.5)	139 (22.0)	93 (14.7)
	HCWs	43 (9.8) [8.5, 11.3]	55 (12.6) [10.9, 14.4]	45 (10.3) [8.9, 11.9]	141 (32.2) [29.8, 34.7]	123 (28.1) [25.4, 30.9]	31 (7.1) [5.9, 8.5]
A COVID-19 vaccine should be given to people who are more at risk for severe illness, only[2]	CMs	27 (4.3)	48 (7.6)	108 (17.1)	177 (28.1)	153 (24.3)	118 (18.7)
	HCWs	13 (3.0) [2.2, 4.0]	33 (7.5) [6.2, 9.1]	65 (14.8) [12.8, 17.2]	135 (30.8) [28.4, 33.4]	136 (31.1) [28.1, 34.1]	56 (12.8) [11.0, 14.8]
A COVID-19 vaccine should be given to everyone, not just those who are more at risk for severe illness	CMs	174 (27.6)	114 (18.1)	108 (17.1)	74 (11.7)	61 (9.7)	100 (15.6)
	HCWs	152 (34.7) [31.6, 38.0]	111 (25.3) [23.1, 27.7]	48 (11.0) [9.1, 13.1]	46 (10.5) [9.1, 12.0]	27 (6.2) [5.1, 7.4]	54 (12.3) [10.8, 14.1]

[1] 95% CI presented for HCWs as survey methods were used for HCW data analysis based on health facility clustering.
[2] For example: elderly, health and other essential workers, people with chronic health conditions etc.

Supplemental Table S2. General vaccine confidence among community members (N=631) and healthcare workers (N=438), North Kivu, The Democratic Republic of the Congo, 2021.

Item	Respondent Group	Very much	Somewhat	Very Little	Not At All	Unsure / Declined
				n(%) [95% CI]*		
How much do you think that vaccines are good?	CM	240 (38.0)	220 (34.9)	72 (11.4)	40 (6.3)	59 (9.4)
	HCW	258 (58.9) [55.7, 62.1]	130 (29.7) [27.1, 32.4]	27 (6.2) [5.1, 7.4]	7 (1.6) [1.1, 2.4]	16 (3.7) [2.8, 4.8]
How much do you think that vaccines are safe?	CM	170 (26.9)	209 (33.1)	33 (21.1)	54 (8.6)	65 (10.3)
	HCW	190 (43.4) [40.5, 46.3]	168 (38.4) [35.8, 41.0)	36 (8.2) [6.9, 9.7]	17 (3.9) [3.0, 5.0)	27 (6.2) [5.0, 7.5]
How much do you think that vaccines protect against diseases?	CM	255 (40.4)	233 (36.9)	77 (12.2)	25 (4.0)	41 (6.5)
	HCW	256 (58.4) [55.5, 61.3]	129 (29.5) [27.0, 32.0]	27 (6.2) [5.1, 7.5]	14 (3.2) [2.4, 4.2]	12 (2.7) [2.1, 3.6]
To which extent do religious leaders in your community approve of vaccination	CM	314 (49.8)	154 (24.4)	50 (7.9)	14 (2.2)	99 (15.7)
	HCW	236 (53.9) [50.8, 56.9]	106 (24.2) [21.9, 26.7]	25 (5.7) [4.3, 7.5]	14 (3.2) [2.4, 4.2]	57 (13.0) [10.9, 15.5]
How much do other leaders in this community approve of vaccination?	CM	290 (46.0)	186 (29.5)	42 (6.7)	17 (2.7)	96 (15.2)
	HCW	228 (52.1) [49.1, 55.0]	118 (26.9) [24.6, 29.3]	21 (4.8) [3.8, 6.0]	11 (2.5) [1.9, 3.4]	60 (13.7) [11.4, 16.4]
		**Positively**	**Mixed**	**Negatively**	**-**	**Don't Know/ Declined**
How do people in this community usually speak about vaccination?	CM	84 (13.3)	354 (56.1)	136 (21.6)	-	57 (9.0)
	HCW	45 (10.3) [8.5, 12.3]	267 (61.0) [58.2, 63.7]	109 (24.9) [22.5, 27.5]	-	17 (3.9) [3.0, 4.9]
				Median [IQR]		
Total Vaccine Confidence Composite Score	CMs			12 [9–15]		
	HCWs			14 [11–16]		

Supplemental Table S3. Information important for vaccine-related decisions among community members (CMs) and healthcare workers (HCWs), North Kivu, The Democratic Republic of the Congo, 2021.

	CMs		HCWs	
	**n (N=631)**	**%**	**n (N=438)**	**% (95% CI)[1]**
**What information is important to help you decide to get vaccinated?[2]**				
Side Effects	285	45.2	209	47.7 (44.4, 51.0)
Cost	25	4.0	22	94.9 (93.7, 96.0)
Location where vaccine is offered	117	18.5	96	21.9 (19.6, 24.4)
Vaccine efficacy	404	64.0	318	72.6 (69.8, 75.2)
Eligibility information	201	31.9	209	47.7 (44.4, 51.0)

[1] 95% CI presented for HCWs as survey methods used for HCW data analysis
[2] Multiple selections allowed therefore proportions do not sum to 100%

Abbreviations: CI, confidence interval
